# Good physicians from the perspective of their patients

**DOI:** 10.1186/1472-6963-4-26

**Published:** 2004-09-12

**Authors:** Ami Schattner, Dan Rudin, Navah Jellin

**Affiliations:** 1 Department of Medicine, Kaplan Medical Center, Rehovot, Israel; 2 Hebrew University and Hadassah Medical School, Jerusalem, Israel; 3 Division of Biostatistics, Clalit Health Services, Tel Aviv, Israel

## Abstract

**Background:**

It is not currently known what is the patient's viewpoint of a "good" physician. We set out to define patient's priorities regarding different physician's attributes in 3 domains important in medical care.

**Methods:**

Patients hospitalized or attending clinics at a large teaching hospital selected the 4 attributes that they considered most important out of 21 listed arbitrarily in a questionnaire. The questionnaire included 7 items each in the domains of patient autonomy, professional expertise and humanism.

**Results:**

Participating patients (n = 445, mean age 57.5 ± 16 years) selected professional expertise (50%), physician's patience and attentiveness (38% and 30%, respectively), and informing the patient, representing the patient's interests, being truthful and respecting patient's preferences (25–36% each) as the most essential attributes. Patient's selections were not significantly influenced by different demographic or clinical background. Selections of attributes in the domain of patient's autonomy were significantly more frequent and this was the preferred domain for 31% and as important as another domain for 16% – significantly more than the domain of professional expertise (P = 0.008), and much more than the domain of humanism and support (P < 0.0005).

**Conclusions:**

Patients studied want their physicians to be highly professional and expert clinicians and show humaneness and support, but their first priority is for the physician to respect their autonomy.

## Background

An expert clinician whose choices are evidence-based; sensitive and dedicated to the patient – not just disease-oriented; who heeds and respects the patient's autonomy, striving at conveying all relevant information to the patient to enable a truly shared decision-making. That can be seen as a prototype or role model of a first class physician. However, these laudable qualities, discussed in many texts [1-3], were purely conceived by physicians, for physicians. How do *patients* value these different physicians' qualities? What would *their* priorities be? This unique point of view, little discussed in previous studies, is the subject of our current research.

## Methods

Patients hospitalized at our 600-bed teaching medical center or attending one of its day clinics were randomly approached and asked to fill in a one-page questionnaire. Over a period of one month, patients were approached by one of the authors (DR) and invited to participate. Every third patient on the specific day's ambulatory clinic lists and every fifth patient on the list of patients hospitalized at the Department of Medicine were approached. If the fifth patient was found to be too ill to participate (e.g. ventilated, unstable or confused) the next name on the list was selected. These numbers were arbitrarily chosen. Following a brief explanation they were handed the questionnaire. The questionnaire listed 21 physicians' characteristics or behaviors regarding the care of patients, presented in a varying order (different order for different patients) to prevent bias due to an item's position on the list. Patients were asked to select 4 attributes that they considered the **most **important and would like the **best **in their own physician. No grading was required. The questionnaire aimed at the patient's image of an excellent physician in general, and did not specify whether the physician was hospital-based or in primary care. The 21 attributes included 7 characteristics in each one of three domains: reflecting professional expertise and high-quality care; reflecting a humanitarian, patient-centered approach; and reflecting patient's autonomy and attentiveness to the patient's preferences and rights. Then patients supplied basic demographic data and the questionnaire was collected about 20 minutes later. The selection of the 21 different physician characteristics or behaviors started from collecting pertinent articles on patient autonomy; physician's humanism and patient support; and on physicians' expertise and professionalism using the author's (AS) collection and a Medline search. Some of the articles were based on patient-derived data. The next stage involved a series of meetings and discussions at the Department of Ambulatory Care and Prevention, Harvard Medical School, Boston. Three senior researchers (2 clinicians, all with extensive experience in research and medical education) – RH Fletcher, T Peters and AS, selected and categorized the 21 items based on the literature and on personal experience. The third stage was a validation study among thirty residents in various stages of their training. They were presented with a mixed list of the 21 items and requested to categorize each one into one of the 3 domains. To rule out that patients categorized these characteristics differently, we performed an additional post-study validation on 30 outpatients that were representative of our study population. Results were similar to those obtained from the residents and confirmed that the vast majority of participants view each of the items as representative of the corresponding domain (Appendix 1) [see additional file 1]. No changes in classification were necessary following the validation study. We planned to a) quantify and study the most 'popular' physician's attributes selected by patients as well as those selected by only a few patients. b) find-out for each patient, whether any one domain was over-represented in the patient's selections (e.g. 2 out of 4 selections belonging to a single domain and less than 2 for each of the others) or – under-represented (no selections in a domain). The study was approved by our Institutional Review Board. Statistical analysis was done using chi-square tests to examine differences between the domains, and between preference of each domain and demographic variables. T-test or one-way ANOVA were used when appropriate.

## Results

A total of 450 patients received the questionnaire and all but 5 consented to participate and returned the filled questionnaires (n = 445). Patient's ages varied from 18 to 89 years (mean 57.5 ± 16) and two thirds were aged 50–80 years. Other patient's characteristics are summarized in Table 1. The top eight physician's qualities preferred by the patients participating in the study (each selected by >25% of patients) are given in Table 2. (Top) and the five physician's qualities selected by <5% are given in the bottom of the same Table. When we transposed each attribute for the domain it represents, basically 2 types or patterns of responses were generated: the AAPH type, reflecting this patient's preference for domain A; and the AAPP type, reflecting the importance (but not dominance) of domain A (as well as P). When no selection at all was made in one domain (such as domain H in the AAPP example), this was also noted (Table 3). Analysis of patients' preferences of physicians' qualities according to domains (qualities within Professional, Humanitarian or patient's Autonomy domains), yielded highly significant differences. For example, 139/445 patients (31%) selected more answers in the patient's autonomy domain than in any other domain, and 69 more patients gave equal importance to patient's autonomy and to one other domain (mostly professionalism). In contrast, humanistic qualities of physicians were selected as the most important by 76/445 patients (17%) only, and just 48 additional patients gave an equal importance to the humanistic and one other domain. Altogether, 90/445 patients clearly selected more characteristics in the professional domain than in any other domain (20%), and 86 other patients gave equal importance in their choices of qualities to the professional and one other domain. Thus, 69% of participating patients gave clear indication as to their preferred domain of physician's characteristics in administering medical care (Table 3). When these preferences and those of patients who entirely disregarded the domain were analyzed and compared, significant differences were found favoring the domain of patient's autonomy (P = 0.008 vs. professional expertise and P < 0.0005 vs. humane attitude) (Table 3). About 10% (38/445), selected equally between the three domains (i.e. one quality of each domain). The remaining 102/445 (22%) were 'indeterminate' in that their choices gave equal importance to two domains. When we analyzed patient's responses to the most essential questions in each of the domains, a similar pattern emerged. For example, 34% selected 2 or more qualities pertaining to patient's autonomy, and 21% selected none. In comparison, <10% named = 2 'humanistic' qualities and over 50% selected none (P < 0.0005). When patient's preferences of the different physicians' characteristics or domains were further analyzed according to the patient's age, gender, origin, income, being hospitalized or main diagnosis – no statistically significant differences or associations could be identified (not shown).

## Discussion

Our study population of 445 patients was heterogeneous (Table 1), yet no statistically significant relationship could be demonstrated between demographic or clinical variables and patient's choices or priorities. These facts lend more impact to our findings.

Patient's preferences of physician's attributes were found to be as unique and individual as the patients themselves. It is remarkable that among 445 patients, only 6 made identical choices (1%). Not surprisingly, patients want their physicians to be experienced and highly professional. This was the physician's characteristic that was chosen by 50% of the patients (Table 2, Top). However, 4 of the other 7 most frequently selected attributes, each selected by 25–38% of the patients, were in the domain of patient's autonomy (523/1172 responses, 45%). The distinct priority accorded by patients to attributes in the realm of patient's autonomy, overrides even the domain of professionalism, and certainly that of physician's humanism and support. Attributes belonging to the domain of patient's autonomy were uncommon among the least demanded attributes (1 of 5, Table 2, Bottom), and were significantly more often selected and less often disregarded than any other domain (Table 3). In contrast, attributes of humanism were not selected *at all *by almost 30% of participating patients. An overview of the results reveals that patients studied want their physicians to show professional expertise and provide humane personal care (a preferred or important domain for 39% or 28% of the patients, respectively) (Table 3) – however, it is even *more *important to them to be well informed and participate in decisions (Tables 2, Top, and 3).

On the other hand, 'humane' qualities that are traditionally considered important, such as showing empathy or being friendly with the patient were surprisingly found to be among the least selected attributes (3–4%, Table 2, Bottom). Moreover, patients participating in this study seldom selected professional qualities such as research and teaching abilities, although they often go hand in hand with appointments at academic medical centers and high quality medical care [4]. Patients also seemed unaware of the unequivocal power of the prevention of "accidents waiting to happen" [5] that can be offered to them by professional physician's counsel. Only 69 patients (15.5%) selected this option as one of four of their priorities. What patients did value, in addition to clinical experience and being up-to-date (Table 2, Top), was for their physicians not to be impatient (38%) or distracted (30%). These two attributes, perhaps the most vulnerable to current time constraints in clinical practice, may reflect patient's needs of a more relaxed, leisurely communication with their physician, be it at the hospital or in primary care. This issue may be more pertinent with the recent changes in context of the consultation, mandating more informed patients and shared decision-making [6,7].

The reason why patient's autonomy features so highly in our study, is not entirely clear. The patient population was heterogeneous (Table 1) so that factors related to origin, income or education are unlikely to be operative. Our medical center is serving a large (about 250,000) population in central Israel which is mostly urban but also rural, and has nothing unique regarding physicians or health delivery system. It is conceivable that the growing emphasis in recent years on patient's rights and patient's autonomy which is prominently reflected in the media and draws much public attention, affected patient's preferences. Further studies will be needed to determine whether, as we believe, our results reflect a novel worldwide trend in patient's preferences. Several limitations of our study ought to be considered. The study was performed in Israel, on a predominantly Jewish population. Thus, its generalizability is open to question. However, Jewish medical ethics put no special value on patient's autonomy so that the findings are likely to apply to other Western affluent societies as well. Also, intentionally, our methodology does not address the relative weight of respondent opinion. Thus, it is likely – but uncertain – that choosing more attributes in a domain is the crucial measure of the relative importance of a domain. Finally, we cannot be entirely certain that the selection process did not lead to some bias, although we consider this contingency to be unlikely.

The age of paternalism in medical care has come to an end and few are sorry for its demise. Most patients want to be informed about their health even if the news are bad [8], and to be involved with their care plans [9]. To do that, patients must have clear information, which takes into account their unique circumstances [10], and there is no better source for that than the patient's physician. Our results strongly suggest that patients expect their physicians to heed these needs and prefer physicians who are sensitive to the varied aspects of patient's autonomy and patient's rights (Appendix 1) [see additional file 1]. The studies of Thom et al. have already indicated that certain physician's behaviors were important for patient's trust. Prominent among them were discussing options with the patient and finding out preferences – essential components of patient autonomy [11]. Similar to the present study, measures of professional competence and humanism were also required. Patient trust was significantly correlated with compliance and with clinical improvement [12]. Since patient trust appears to be such a crucial component of the patient-physician relationship, and since trust is dependent on patient's preferences being met [12] it is mandatory to establish what these preferences are today, and this is where our study comes in.

Previous research has already identified the complexity of patient's needs in the modern era. While older studies make no mention of issues of patient autonomy [13], a relatively recent systematic review of the literature on patient's priorities found "humaneness" to be the most highly rated aspect of care, followed by clinical competence and patient's participation in decisions [14]. Another study from Scotland identified physicians' attentiveness and patience at the top of the list, and patients in the Netherlands mostly desired sufficient consultation time [15,16]. However, a strong desire for information and participation in decision-making already features in these and other studies [14-18]. Among our patients it came out for the first time as a top priority, second to none (Table 3). Failure of physicians to provide a patient-centered approach may therefore seriously undermine patient's expectations and satisfaction. Recent data suggest that this may be associated with significant adverse outcomes [19,20]. Physicians can be effectively trained to listen to the patient's narrative [21], recognize the patient's perspective [7,22] and adopt a more patient-centered approach [23]. However, besides skills in communication [21,24,25], this requires time. Time management can also be effectively taught [25] but it is still unclear whether decreasing hospital length of stay and consultation time in primary care will not adversely affect physicians' performance and patient's expectations [3]. Nevertheless, patient's preferences remain integral to modern evidence-based practice [26], and our study should provide a poignant reminder that autonomy is nowadays what the patients want most.

## Conclusions

Our study focused on the selections of several hundred outpatients and inpatients, regarding their preferences for different attributes of their physicians. We found heterogeneous preferences, but attributes in the domain of patient autonomy and physicians' expertise (in that order), headed the list.

## Competing interests

None declared.

## Authors' contributions

AS initiated, designed and supervised the study, analyzed the results and wrote the manuscript. DR performed the actual study. NJ provided statistical advice and analysis. All authors read, discussed, contributed to and approved the final manuscript.

## Pre-publication history

The pre-publication history for this paper can be accessed here:



## Supplementary Material

Additional File 1 The patient's questionnaire including the 21 attributes in the three domains. Click here for file

## Figures and Tables

**Table 1:** Characteristics of 445 patients participating in the study (%).

**Characteristic**		**Percentage**
Gender	Male	46
	Female	54
Origin	Ashkenazi	31
	Sephardic	22
	Israeli	47
Family status	Married	75
	Single	9
	Divorced	6
	Widower	10
Income	High	18
	Medium	60
	Low	22
Setting	Ambulatory	77
	Hospitalized	23
Main diagnosis	Cardiovascular	24
	Gastroenterological	14
	Pulmonological	12
	Dermatological	11
	Endocrinological	10
	Rheumatological	8
	Neurological	8
	Oncological	5
	Nephrological	4
	Hematological	4

**Table 2:** The eight 'best' physicians' qualities preferred by more than a quarter of the patients (Top) and the least desired physicians' qualities, each preferred by less than 5% of the patients (Bottom).

Physicians' attribute / Domain**	**Patients' preference (Number, %)**
**Most desired**	
Experienced [Prof.]	223 (50%)
Patient [Hum.]	170 (38%)
Informs the patient [Auton.]	159 (36%)
Attentive [Hum.]	132 (30%)
Represents patient's interests [Auton.]	127 (29%)
Truthful [Auton.]	125 (28%)
Up-to-date [Prof.]	124 (28%)
Respects patient's preferences [Auton.]	112 (25%)
**Least desired**	
Discretion [Auton.]	22 (5%)
Empathy [Hum.]	19 (4%)
Research [Prof.]	17 (4%)
Friendly [Hum.]	12 (3%)
Teaching [Prof.]	3 (1%)

** Auton. = patient's autonomy and patient's rights; Prof.= Professional expertise; Hum.= humanism and support (Detailed in the Appendix [see additional file 1]).

**Table 3:** Patient's priorities according to domains (n = 445), number (%).

	**Patient's autonomy**	**Professional expertise**	**Humane attitude**
**Preferred domain ^#^**	139 (31)	90 (20)	76 (17)
**Important domain^##^**	69 (16)	86 (19)	48 (11)
**Combined ^###^**	47%	40%	28%
**No choices in the domain**	54 (12)	64 (14)	119 (27)
**Significance**	P = 0.008 vs. Prof P < 0.0005 vs. Hum.	P < 0.0005 vs. Hum.	

# Preferred domain = at least 2 choices (a majority) of attributes in that domain and more than in any other domain.

## Important domain = domain selected as important, but with equal selections in one other domain. (In an additional 9% of the patients, all 3 domains were equally selected).

### Combined 'preferred' and 'important' domains.
