# Supplementary material for: Good physicians from the perspective of their patients
Source: BMC Health Serv Res. 2004 Sep 12;4:26. doi: 10.1186/1472-6963-4-26 (PMC520754; doi:10.1186/1472-6963-4-26)
Supplement: Additional File 1 — The patient's questionnaire including the 21 attributes in the three domains. [file 1472-6963-4-26-S1.doc]

**Appendix 1.**

The patient’s questionnaire including the 21 attributes in the three domains @

“Please identify the 4 **most essential attributes** that you would like most to find in **your** doctor, and that **you believe are more important** than the rest.”

**I. Patient’s autonomy and patient’s rights domain**:

1. Finds out what is important for you and takes your preferences into consideration.
2. Provides a clear explanation of the disease, elaborating on treatment options and possible adverse outcomes.
3. Continues to take care of you on different visits.
4. Tells you the whole truth about your condition and treatment.
5. Asks your own opinion as to what may be wrong with you.
6. Gets for you the tests or treatments you need despite difficulties or cost.
7. Can be completely trusted with guarding your secrets and being discrete.

**II. Professional expertise domain**:

1. Is very experienced and professional.
2. Is current with important new developments in medicine.
3. Will readily consult other physicians in case of a problem.
4. Carefully considers and reviews each option and decision.
5. Considers not only your current chief complaint but also suggests effective preventive measures.
6. Is also a scientist with a record of original research and publications.
7. Is a competent teacher to medical students and junior doctors.

**III. Domain of humanism and support**:

1. Is very attentive to what you have to say.
2. Is not distant or arrogant but treats you as an equal.
3. Shows patience and devotes enough time to you.
4. Provides encouragement and hope.
5. Is friendly, informal and jokes with you.
6. Knows you and your family personally.
7. Is sensitive to your feelings and shows empathy.

@ In the actual questionnaire, the domains were not defined and the 21

attributes were presented in a different order for different patients, to avoid bias. The attributes were defined and validated in a preliminary study (see text).
